# Supplementary material for: Resolving the full spectrum of human genome variation using Linked-Reads
Source: Genome Res. 2019 Apr;29(4):635–45. doi: 10.1101/gr.234443.118 (PMC6442396; doi:10.1101/gr.234443.118)
Supplement: Supplemental Material [file supp_gr.234443.118_Supplemental_longranger-2.2.zip › longranger-2.2.tar.bz2!/lr2.2-public/lib/stan/cmdstan/stan/doc/api/html/math_2functions_2fdim_8hpp_source.html]

Stan: src/stan/math/functions/fdim.hpp Source File


|  |  |
| --- | --- |
|  | Stan  2.5.0  probability, sampling & optimization |


- Main Page
- Related Pages
- Namespaces
- Classes
- Files

- File List
- File Members

All Classes Namespaces Files Functions Variables Typedefs Enumerations Enumerator Friends Macros Pages

- src
- stan
- math
- functions

fdim.hpp

Go to the documentation of this file.

1 #ifndef STAN\_\_MATH\_\_FUNCTIONS\_\_FDIM\_HPP

2 #define STAN\_\_MATH\_\_FUNCTIONS\_\_FDIM\_HPP

3

4 #include <math.h>

5 #include <boost/math/special\_functions/fpclassify.hpp>

6 #include <boost/math/tools/promotion.hpp>

7

8 namespace stan {

9  namespace math {

21  template <typename T1, typename T2>

22  inline typename boost::math::tools::promote\_args<T1, T2>::type

23  fdim(T1 a, T2 b) {

24  if (boost::math::isnan(a) || boost::math::isnan(b))

25  return std::numeric\_limits<typename boost::math::tools::promote\_args<T1, T2>::type>

26  ::quiet\_NaN();

27  return ::fdim(a, b);

28  }

29  }

30 }

31

32 #endif

boost::math::isnan

bool isnan(const stan::agrad::var &v)

Checks if the given number is NaN.

**Definition:** boost\_fpclassify.hpp:69

stan::math::fdim

boost::math::tools::promote\_args< T1, T2 >::type fdim(T1 a, T2 b)

The positive difference function (C99).

**Definition:** fdim.hpp:23


---

[ Stan Home Page ]

*© 2011–2014,
Stan Development Team.*
